# Chronic Urticaria: Indian Context—Challenges and Treatment Options

**DOI:** 10.1155/2013/651737

**Published:** 2013-09-24

**Authors:** Sujoy Khan, Anirban Maitra, Pravin Hissaria, Sitesh Roy, Mahesh Padukudru Anand, Nalin Nag, Harpal Singh

**Affiliations:** ^1^Consultant Allergist & Immunologist, Department of Allergy & Immunology, Apollo Gleneagles Hospital, 58 Canal Circular Road, Kolkata, West Bengal 700 054, India; ^2^Department of Paediatric Pulmonology, Vision Care Hospital, Mukundapur, Kolkata West Bengal 700099, India; ^3^Department of Allergy, Immunology and Arthritis, Apollo Hospitals International Ltd., Bhat GIDC, Gandhinagar, Ahmedabad, Gujarat 382428, India; ^4^Dr. Niphadkar's Asthma and Allergy Health Clinic, Hindu Colony Lane No. 1, Dadar (East), Mumbai 400 014, India; ^5^Department of Pulmonary Medicine, JSS Medical College, JSS University, Mysore, Karnataka 570 004, India; ^6^Department of Medicine, Indraprastha Apollo Hospital, Mathura Road, Jasola Vihar, New Delhi 110076, India; ^7^Clinical Marketing Manager, Phadia/IDD, Thermo Fisher Scientific, Units No. 7, 10 & 11, Splendor Forum, Plot No. 3, Disttrict Centre, Jasola, New Delhi 110025, India

## Abstract

Urticaria is a common condition that occurs in both children and adults. Most cases have no specific allergic trigger and the aetiology of urticaria remains idiopathic and occasionally spontaneous in nature. Inappropriate advice such as avoidance of foods (milk, egg, prawn, and brinjal) is common place in certain sections of India mostly by nonspecialists that should not be routinely recommended. It is important to look for physical urticarias such as pressure urticaria in chronic cases, which may be present either alone or in combination with other causes. Autoimmune causes for chronic urticaria have been found to play an important role in a significant proportion of patients. Long-acting nonsedating antihistamines at higher than the standard doses is safe and effective. Quality of life is affected adversely in patients with chronic symptomatic urticaria and some may require multidisciplinary management.

## 1. Background

Urticaria is a common condition and the chronic form usually has no allergic trigger. Longacting non-sedating antihistamines at higher than the standard doses is safe and effective.

Urticaria is characterized by itchy, red, raised (wheal), and flared skin reactions that last usually for a few hours (typically <24 hours). It is classified as chronic urticaria (CU) if it lasts for more than 6 weeks. The chronic spontaneous form of urticaria does not need any stimulus and sometimes it is also referred to as chronic idiopathic urticaria (CIU) [1–6]. It is now well recognized that CIU consists of a myriad group of diseases and development of skin lesions and/or angioedema is seen in all different types and subtypes [4–6]. The terms CIU and CU have been used interchangeably in the article, although strictly CIU would refer to patients without a proven autoimmune component to the urticaria [5–9].

The wheal has a central swelling surrounded by a reflex erythema that is itchy while the angioedema is associated with pronounced swelling of the lower dermis and subcutaneous tissue with the occasional involvement of mucous membranes (lips, tongue) in some patients. Acute urticaria appears more commonly in children and young adults of which common causes are infections, food, drugs (intravenous more than oral forms), and insect stings. It is important to take a detailed clinical history to identify whether the urticaria is chronic (or acute or chronic), as occasionally a patient may be wrongly labeled as drug allergic when it may be that the urticaria was present *before* the drug was started [1–3]. There are some drugs, however, that are notorious in causing urticaria due to a nonspecific mast cell stimulation such as opiates, high-osmolar radiocontrast media, and vancomycin. A physical examination (combined with history taking) is important as the diagnosis of urticaria remains a clinical one, apart from a few supportive investigations that could only label the cause as autoimmune.

This paper aims to discuss existing guidelines of urticaria in the Indian context, with an attempt to demystify some of the myths surrounding this condition based on our collective experience and extensive publications in this field. This paper is, therefore, applicable or relevant to physicians working in India or South East Asia where nonspecialists deal with the majority of cases of urticaria, and higher specialist training in the field of Allergology is yet to begin. This article does not aim to review the urticarias but to discuss the current level of understanding of the patients and treatment options (feasible and otherwise) to the physicians.

## 2. Consensus Guidelines on Urticaria

The EAACI/GA²LEN/EDF/WAO consensus guideline for the diagnosis and management of urticaria was published in 2009. These were based on expert recommendations from the Third International Consensus Meeting on Urticaria (Urticaria 2008), joint initiative of the EAACI Dermatology Section, Global Allergy and Asthma European Network (GA²LEN), European Dermatology Forum (EDF), and World Allergy Organization [1, 2]. Since then, several other societies have also published guidelines but have essentially maintained the messages of the 2009 guidelines. The important messages for clinicians and researchers in this field were (1) the absence of reliable assessment tools including specific laboratory markers and (2) the absence of effective long-term treatments for this common condition. A subsequent update from the GA²LEN task force also identified several unmet clinical needs in patients with chronic spontaneous urticaria [3]. 

The worldwide incidence is 0.1%–3% of the population with women affected twice more likely than men. It is estimated that about 1 in 5 people will have urticaria once in their lifetime and this seems to be the case across all age groups. Up to 1% of the population suffers from chronic urticaria (CU) and all age groups appear to be affected, although the peak incidence is between 20 and 40 years of age. In most cases, the disease lasts between 1 or 5 years, but the duration can be longer for those with severe urticaria, those with concurrent angioedema, those with the physical component, and those with a positive autologous serum skin test.

Although up to half of the patients with CIU have an IgG autoantibody directed against the alpha subunit of the high-affinity IgE receptor (Fc*ε*R1*α*) which is believed to be the pathophysiological basis of autoimmune urticaria, the role of antithyroid antibodies on persistent cutaneous mast cell and basophil activation remains unproven [5–9]. The role of the coagulation cascade (particularly the extrinsic pathway) is interesting as patients with severe disease have an increased thrombin generation; higher fragment F(1+2), D-dimer, and activated factor VII plasma levels, while increasing tissue factor reactivity in the skin. Takeda and colleagues showed that levels of fibrinogen, D-dimer, fibrin and fibrinogen degradation products were significantly raised in CU patients with a hypercoagulable state on APTT waveform analyses [10]. It is, therefore, not surprising that acute phase reactants like C-Reactive Protein (CRP) and procalcitonin levels are raised in patients with severe CU as compared to healthy controls or mild CU patients, including several other cytokines [11–24] and the soluble serum factor that leads to the release of histamine from basophils [25, 26]. Although histamine plays a significant role in diseases like CU and eczema, prostaglandins, leukotrienes (LTs), and cytokines such as IL-31 seem to prolong the inflammatory process. 

## 3. Myths about Urticaria and Reality

*Myth 1*. Patients with urticaria have multiple allergies.

*Reality*. Most patients with urticaria do not have allergies, and patients who have positive specific IgE to allergens usually do not find any objective improvement on the avoidance of such allergens [27]. It is well accepted that a very high total IgE (usually a feature of atopy but also seen in some patients with urticaria) leads to low-level “false-positive” specific IgE results. Clinicians need to consider this before interpreting the results and advising patients to avoid multiple “triggers” for the urticaria. It is, therefore, not useful to do IgE levels in patients with only CU as it does not affect the management plan.

*Myth 2*. Patients with urticaria should be given an extensive list of foods that must be avoided.

*Reality*. Our collective experience has shown that patients are often asked by nonspecialists to avoid egg, milk, brinjal, spinach, prawn, and fish as these are the “triggers” for urticaria. Strict avoidance has little or no effect on the frequency of urticarial eruptions. There are some foods that do, however, have or can release more histamine and clinical advice often entails educating patients to avoid eating most of the foods that are high in histamine during acute urticarial eruptions until the “episode” settles down. Skin prick testing to these foods in patients with chronic urticaria does not show any wheal or flare response suggesting the absence of a specific IgE or the supposed “trigger” factor(s). 

A small cohort of paediatric patients with CU underwent skin testing to foods that were being avoided based on ELISA allergy results at one centre (further details with Dr. Sujoy Khan, Apollo GleneaglesHospital, Kolkata). None of the 30 children with CU (mean (±SD) age was 10.9 (±4.2) years, 13 males and 17 females) demonstrated skin test reactivity to milk, egg white, egg yolk, prawn, brinjal, and spinach which were the foods on the exclusion list. All patients were able to resume a normal diet on high dose antihistamines that controlled the urticaria. 

In selected patients with supportive histories, presence of IgE to specific foods or sensitivity (non-IgE mediated reactions) to certain dyes or coloring agents in food (pseudo allergies) could have a relevance to their chronic urticaria symptoms, but careful elimination and reintroduction are needed to establish the same [28]. 

*Myth 3*. Patients with urticaria should undergo testing to exclude specific allergies.

*Reality*. Whilst the concurrent presence of house dust mite (*D. pteronyssinus, D. farinae,* and *Blomia sp.*) allergy or other aeroallergen sensitivities can be found in some patients [27, 29–31], these tests should be reserved for patients who complain of allergic rhinitis symptoms that occur without urticaria. 

In view of this perception that allergy testing is mandatory, another observational study at one centre on 43 consecutive patients with chronic urticaria (dermographism, autoimmune thyroiditis excluded) with skin prick testing to aeroallergens was carried out (further details with Dr. Sujoy Khan, Apollo Gleneagles Hospital, Kolkata). Skin prick tests (SPT) were done after a 7-day antihistamine free period to house dust mites (*Dermatophagoides pteronyssinus*, *Dermatophagoides farinae*, and *Blomia tropicalis*), cockroach, pollens, moulds, and animal dander in all patients. Positive control was histamine (10 mg/mL) and positive SPT was defined as >3 mm than the negative control (saline). 

Nonparametric statistical data were calculated using the GraphPad Prism software Version 5.04 (GraphPad Software, Inc., La Jolla, CA, USA). Fisher's exact test was used to see the relationship between CIU, mite reactivity status, and with/without respiratory symptoms (allergic rhinitis, asthma).

The mean (±SD) age was 33.28 (±14.97) years that included 23 males and 20 females. Range of duration of the symptoms of CIU was 6 months to 13 years. SPT demonstrated immediate reactivity to dust mite in 24 patients (55.8%), cockroach 6 (14%), pollens 8 (18.5%), moulds 5 (11.6%), and dander 0 (0%). Five patients were polysensitized (dust mites, cockroach, pollens, or moulds). The mean (±SD) age of patients with CIU and mite allergy was 31.1 (±14.7) years compared to 36.1 (±15.2) years in CIU patients without mite allergy (nonsignificant, 2-tailed *t*-test 0.2849). Among mite positive patients with CIU, there was a slight female predominance (13 females, 11 males) that was statistically nonsignificant (*P* = 0.3586). However, 16 CIU patients with respiratory symptoms had dust mite reactivity as compared to 3 CIU patients with respiratory symptoms but without mite reactivity (highly significant, *P* = 0.0016). 

We conclude from this study that house dust mite reactivity in CIU is linked with respiratory allergy. Avoidance of these allergens will, therefore, have little effect on urticaria, except in a few cases where there is a strong consistent history of contact urticaria on exposure to dust, but antihistamine and nasal spray treatment will have an effect on the rhinitis and will encourage the patient to continue on antihistamines that will control the urticaria. Routine skin prick testing or specific IgE allergy tests when no trigger is identified on history taking cannot be recommended. Again, in some highly atopic individuals, allergens such as grass pollens, molds, animal dander, house dust mites, and latex might aggravate chronic urticaria but this is usually not the primary cause for the urticaria.

*Myth 4*. Patients should not receive high doses of antihistamine medications and definitely not in pregnancy.

*Reality*. Almost all physicians dealing with CIU patients recognize that standard or recommended doses of antihistamines are ineffective in treating this condition. Consensus guidelines do take this seriously and specifically comment that higher doses, even up to four fold higher, are safe and have been verified in studies [1–6]. This is true for all classes of antihistamines such as desloratidine, levocetirizine, fexofenadine, and even antiplatelet activating factor blocker rupatadine. First generation antihistamines should preferably be avoided in infants and children as well as adults especially those dealing with heavy machinery or engaged in skilled tasks such as driving. At least two long-term studies on healthy volunteers have shown that fexofenadine at 240 mg once daily for a year is safe, well tolerated, and does not lead to sedation at these supratherapeutic doses [32, 33]. Cetirizine and levocetirizine have sedative effects in therapeutic and supratherapeutic doses, and it is, therefore, best to check with the patient whether sedation has been a problem in the past.

The EAACI/GA²LEN/EDF/WAO consensus guidelines mention that loratidine and possibly desloratidine are safe in pregnancy but supratherapeutic doses should be carefully considered. The product literature of fexofenadine HCl (Sanofi, Aventis Pharma Ltd., CDS version 5 dated Nov 2006) does not mention pregnancy as a contraindication to its use, other than to use it if the benefit outweighs the potential risks. Cetirizine, loratidine, and hydroxyzine have been shown to be safe in pregnancy with no difference in spontaneous or therapeutic abortions, birth weight, mode of delivery, gestational age, and rate of live births, neonatal distress, and major fetal congenital malformations [34, 35]. 

## 4. Quality of Life

It is now well recognized that patients with CU have a poor quality of life (QoL) (see Table 1, [36–42]). Although not specifically addressed in some studies, the “uncertainty” factor of appearance of skin lesions, especially in social gatherings or workplace, plays an important role in affecting the QoL. Several other issues related to poor QoL would include cost of therapy, fatigue associated with use of antihistamines, and inability to explain the skin lesions that may add to social isolation including frustration in dealing with the chronic condition. 

Several tools are available for assessment of impairment of QoL in patients affected by chronic diseases. QoL studies on patients with psoriasis enabled healthcare providers to understand that there were several areas that required attention apart from simply controlling the disease with multiple medications. Staubach and colleagues in an interdisciplinary interview/questionnaire-based study on 100 CU patients found that significantly low quality of life (functioning and emotions) and psychiatric comorbidity (depression, anxiety, somatoform disorders) made this worse even in those without a formal psychiatric diagnosis [39]. In another study by the same group and of 100 patients with CU who were formally assessed for psychiatric illnesses, nearly half (48%) of the patients had one or more psychosomatic disorders, of which anxiety disorders were predominant followed by depressive and somatoform disorders [42]. As the authors rightly concluded, patients with CSU frequently experience anxiety, depression, and somatoform disorders, that with time become inextricably linked to an increased emotional distress.

Studies on fexofenadine-treated patients (180 mg) have shown significantly greater improvements in mean dermatology life quality index (DLQI) total score than those treated with placebo. These were not only seen in areas such as symptoms and feelings, activities of daily living including less impairment while working, leisure, and personal relationships but also greater improvement in Urticaria Activity Score (wheals and pruritis) when compared with placebo. 

Indeed it is interesting that while this disease itself causes distress, chronic urticaria is also recognized as a stress-vulnerable disease in which psychological stressors can trigger or increase itching. It is suggested that effective management processes should take into account the psychological factors in some of the patients and the treatment regimen should be tailored to the individual patient's needs and circumstances [36, 38, 39, 42].

## 5. Treatment Options

The consensus guidelines have adopted the management of urticaria into (1) avoidance measures and (2) pharmacotherapy nonspecific and specific. The avoidance approach outlines elimination or treatment of eliciting stimulus or cause (such as nonsteroidal anti-inflammatory drug-induced urticaria/angioedema, physical causes, treatment of an infectious trigger, etc.) that is not possible in all cases (i.e., those with CIU) [1–6]. In line with this “infectious trigger,” the approach that is gaining relevance is the consideration of *Helicobacter pylori* induced gastritis and urticaria, and several reports of long-lasting remission of urticaria can be seen in patients after eradication therapy [43–47]. 

The second approach is lowering or inhibiting mast cell mediator release and the most commonly used drugs (nonspecific approach) that inhibit mast cell release are corticosteroids. Continuous or prolonged use with corticosteroids to treat urticaria is not recommended as the risks and long-term side effects outweigh the benefits. Specific treatment approaches involve the use of nonsedating long-acting antihistamine (anti-H1) drugs such as cetirizine, levocetirizine, loratadine, desloratadine, and fexofenadine that provide both antiallergic and anti-inflammatory effects, such as inhibition of cytokines release from basophiles and mast cells as well as reduction of chemotactic activity of eosinophils. Doxepin, a tricyclic antidepressant, is the only agent that blocks both H1- and H2-receptors, and can be useful in the selected patients who experience significant psychosomatic symptoms of depression and anxiety due to the urticaria. 

Table 2 provides a list (not comprehensive) of antihistamine medications available in India, including combination formulations that may not be suitable in all patients although they may prove to be cost-effective. 

Montelukast is an orally active leukotriene receptor antagonist (LTRA) licensed the maintenance treatment of asthma and to relieve symptoms of seasonal allergies. Montelukast binds to and blocks the action of leukotriene D4 (LTD4) on the cysteinyl-leukotriene receptor CysLT1 in the lungs, with almost no interaction with other antiallergy drugs. This reduces the bronchoconstrictive and inflammatory effects of LTD4 in the airways. Other LTs such as LTC4, LTD4, and LTE4 have important roles in the pathophysiologic mechanisms of allergic inflammation after binding to activating receptors, cystenyl-LT1 (CysLT1) receptor and Cys-LT2 receptor. Hence, LTRAs such as montelukast 10 mg once daily or zafirleukast 20 mg twice daily has been employed either as monotherapy or in combination with H1-receptor and/or H2-receptor antagonists, to treat different forms of CU, including cold urticaria, urticaria related to food additives, chronic autoimmune urticaria, steroid-dependent urticaria, and delayed-pressure urticaria, and CIU and dermographism with varying results [48–54]. Our report on montelukast as an added therapy to anti-H1 and anti-H2 blockers showed that it was effective in controlling the urticaria in about 50% of the patients (UK based study). However, we were unable to delineate any specific clinical features (such as age, gender, duration, or severity of urticaria) or laboratory features (such as thyroid autoimmunity, antinuclear antibody positivity, or basophil histamine release potential) that could predict a response to montelukast [54]. 

Other treatment options that have significant activity on mediator release on basophils include the calcineurin inhibitor cyclosporin A [55–60], and occasionally ultraviolet therapy [59–62]. As for immunosuppressive therapy with cyclosporin, a recent study suggests that history of hives, shorter duration of urticaria (mean of 55.2 weeks versus 259.6 weeks, *P* = 0.03), and CU index >10 (*P* = 0.05) predict a favorable response to cyclosporin [60].

The most specific and promising therapy for the future appears to be anti-IgE therapy, Omalizumab (Xolair, Novartis) [63–66]. A typical dose of 150 mg every 2nd/4th week or 300 mg/month for 4–6 doses can have lasting efficacy of up to 15 months with significant improvement in QoL [65–67]. The significant downside is the high cost associated with the treatment (1-2 subcutaneous injections/month at US $10,000/year) and its yet unknown side effects with regard to parasitic infectious disease burden with its use in India or Asia [68–70]. 

## 6. Conclusions

1. Chronic urticaria is a relatively common condition in India and most cases have no specific allergic trigger and remain idiopathic.
2. Autoimmune causes have been found to be associated with up to 30-40% cases.
3. It is important to look for physical urticarias such as pressure urticaria in chronic cases.
4. Avoidance of foods without appropriate testing for food allergy should not be routinely recommended.
5. Long-acting nonsedating antihistamines at even higher than standard doses if necessary are safe and effective.
6. Quality of life is affected adversely in many patients with chronic urticaria.
7. Psychological stressors can play an important role in this disease and require special attention.

## Figures and Tables

**Table 1:** Quality of life (QoL) studies in patients with chronic urticarial.

Study design, place	Methods used	Outcome	Reference
Questionnaire based study on 170 consecutive patients, London (UK)	Dermatology life quality index (DLQI) assessment in different urticarial groups	Moderate impairment in QoL in CU with physical urticaria; significantly higher impairment in patients with DPU and cholinergic urticaria (QoL affected areas: work/study, symptoms/feelings, leisure)	[37]

Interview/questionnaire-based study on 100 in-patients (96 age- and sex-matched controls), University of Mainz (Germany)	Assessed using Skindex-29 on overall QoL and three defined QoL aspects	CU patients had a markedly reduced QoL compared to controls, all 3 areas affected, psychiatric comorbidity was made worse	[39]

Questionnaire based study on 157 CU patients, Berlin (Germany)	CU-QoL, DLQI, and Skindex-29 questionnaires were completed	70% data variance in CU-QoL in functioning, sleep, itching/embarrassment, mental status, swelling/eating, and appearance; sleep and mental health significant areas are affected and women are more affected by pruritis	[41]

Mental disorder assessment on 100 patients, University Medical Center Mainz (Germany)	Specialised diagnostic interviews and psychometric instruments and SCL-90R GSI	48% of patients had one or more psychosomatic disorders; high emotional stress impairing quality of life	[42]

Cross-sectional observational study (*n* = 249), Suwon (Korea)	CU-QoL and UAS; multiple linear regression for CU-QoL predictors	DPU, sunlight exposure, and emotional stress significantly influenced the overall CU-QoL scores (univariate analysis); multivariate regression models indicated that dermatographism and emotional stress were significant predictors of impairment of all four QoL domains	[40]

Abbreviations: QoL: quality of life, CU-QoL: chronic urticaria-quality of life, DLQI: dermatology life quality index, DPU: delayed pressure urticarial, and SCL-90R GSI.

**Table 2:** Overview of medications available in India for urticaria and angioedema.

Type	Generic name	Availability	Price CIMS India*
Antihistamine H1	Cetirizine	Widely available (41 brands)	Re^$^ 1/tablet to Rs 4.75/tab

Antihistamine H1	Chlorphenamine	Widely available (17 brands)	4 mg (500 tablets) from Rs 25.79 to Rs 29.12; Syrup (0.5 mg/5 mL) 60 mL at Rs 23.80

Antihistamine H1	Desloratadine	Widely available (11 brands)	Rs 1.99/tablet to Rs 5.50/tablet

Antihistamine H1	Fexofenadine	Widely available (11 brands)	30 mg 10 tablets for Rs 31.00 120 mg 10 tabs from Rs 40 to Rs 99.15 180 mg 10 tabs from Rs 60 to Rs 111.80

Antihistamine H1	Hydroxyzine	Widely available (4 brands)	10 mg 10 tablets for Rs 9.00 25 mg 10 tablets from Rs 16.00 to Rs 23.00 Syrup (10 mg/5 mL) 100 mL at Rs 40.18

Antihistamine H1	Levocetirizine	Widely available (60 brands)	5 mg 10 tablets from Rs 8.90 to 55.32 Syrup (2.5 mg/5 mL) 30 mL from Rs 18.90 to Rs 29.00

Antihistamine H1	Loratidine	Widely available (6 brands)	10 mg 10 tablets from Rs 19.50 to Rs 150.00 Syrup (5 mg/5 mL) 30 mL for Rs 46.00 Suspension 1 mg 30 mL for Rs 17.85

Leukotriene receptor antagonist (LTRA)	Montelukast Zafirlukast	Montelukast is widely available (14 brands) Zafirlukast is not available	4 mg 10 tablets from Rs 62.50 to 89.00 5 mg 10 tablets from Rs 70 to 98.00 10 mg 10 tablets from Rs 83.20 to 149 Sachet 4 mg 1 sachet costs Rs 5.85

Combinations anti-H1 + LTRA	Levocetirizine 5 mg + Montelukast 4/5/10 mg	Widely available (16 brands)	Rs 5.90 to Rs 16/tablet (adult) Rs 3.80 to Rs 6.30/tablet (kid)

Combinations anti-H1 + LTRA	Fexofenadine 120 mg + Montelukast 4/10 mg	Limited to no availability in smaller cities	10 tablets for Rs 125.00

Anti-PAF	Rupatadine 10 mg	Limited availability (3 brands)	10 mg tablets Rs 5-6/tablet

Combinations anti-PAF + LTRA	Rupatadine 10 mg + Montelukast 10 mg	Limited availability	10 tablets for Rs 84.60 (Rs 8.46/tablet)

Immunosuppressant	Hydroxychloroquine	Widely available	200 mg tablets 10 from Rs 59 to Rs 80

Immunosuppressant	Methotrexate	Widely available	2.5 mg tablets 10 from Rs 15.00 to 57.85

Immunosuppressant	Cyclosporin	Widely available	25 mg tablets from Rs 21.60–32.60/tab 50 mg tablets from Rs 43.20–65.20/tab 100 mg tablets from Rs 82.60–130.40/tab

Anti-IgE	Omalizumab (Xolair, Novartis)	Very limited availability, expensive, and available through select Central Govtenment Health Schemes at reduced costs in India	150 mg injection, frequency, and dose calculated on body weight and IgE level is *₤*256.15 + VAT per vial (NHS, UK) or US$10,000/year (1-2 injections/month)

*Ref: CIMS 115 Oct 2011 (Update-4); costs of the last 4 of 5 drugs were obtained from Medline India.

^$^Indian currency in Rupees (Rs), exchange rate Rs 56.83 = 1 US$ (as on 6 June, 2013).

Abbreviations: PAF: platelet activating factor, LTRA: leukotriene receptor antagonist, IgE: immunoglobulin E, and NHS: national health service.
